# The potential mechanisms of lactate in mediating exercise-enhanced cognitive function: a dual role as an energy supply substrate and a signaling molecule

**DOI:** 10.1186/s12986-022-00687-z

**Published:** 2022-07-30

**Authors:** Xiangli Xue, Beibei Liu, Jingyun Hu, Xuepeng Bian, Shujie Lou

**Affiliations:** 1 Key Laboratory of Exercise and Health Sciences of Ministry of Education, Shanghai University of Sport, Shanghai, 200438 China; 2 Shanghai Frontiers Science Research Base of Exercise and Metabolic Health, Shanghai University of Sport, Shanghai, 200438 China; 3 Department of Clinical Medicine, Weifang Medical College, Weifang, 261053 Shandong China

**Keywords:** Astrocyte-neuron metabolic coupling, Lactate shuttle, Lactate receptor HCAR1/GPR81, Cognitive function, Exercise intervention

## Abstract

Lactate has previously been considered a metabolic waste and is mainly involved in exercise-induced fatigue. However, recent studies have found that lactate may be a mediator of the beneficial effects of exercise on brain health. Lactate plays a dual role as an energy supply substrate and a signaling molecule in this process. On the one hand, astrocytes can uptake circulating glucose or degrade glycogen for glycolysis to produce lactate, which is released into the extracellular space. Neurons can uptake extracellular lactate as an important supplement to their energy metabolism substrates, to meet the demand for large amounts of energy when synaptic activity is enhanced. Thus, synaptic activity and energy transfer show tight metabolic coupling. On the other hand, lactate acts as a signaling molecule to activate downstream signaling transduction pathways by specific receptors, inducing the expression of immediate early genes and cerebral angiogenesis. Moderate to high-intensity exercise not only increases lactate production and accumulation in muscle and blood but also promotes the uptake of skeletal muscle-derived lactate by the brain and enhances aerobic glycolysis to increase brain-derived lactate production. Furthermore, exercise regulates the expression or activity of transporters and enzymes involved in the astrocyte-neuron lactate shuttle to maintain the efficiency of this process; exercise also activates lactate receptor HCAR1, thus affecting brain plasticity. Rethinking the role of lactate in cognitive function and the regulatory effect of exercise is the main focus and highlights of the review. This may enrich the theoretical basis of lactate-related to promote brain health during exercise, and provide new perspectives for promoting a healthy aging strategy.

## Introduction

Population aging has become a worldwide phenomenon. Degenerative changes in brain structure and function occur with aging, causing a cognitive decline in older adults. In addition to aging, vascular dementia, Alzheimer’s disease (AD), Parkinson's disease, Huntington and other neurodegenerative diseases, as well as some metabolic diseases such as diabetes, are accompanied by an increased risk of cognitive impairment or dementia. This poses a great challenge to public health and socio-economic development in many countries. However, due to the difficulties in developing new drugs for cognitive impairment, especially in AD, and the lack of drug-specific therapeutics available for the treatment of AD dementia stage, more and more researchers now focus on non-pharmacological intervention.

As an economical and practical non-pharmacological therapy with no toxic side effects, regular exercise was proved to be an effective means for preventing and treating cognitive impairment or dementia, which could reduce the risk of dementia in healthy people and even higher risk gene carriers. The comprehensive health benefits of exercise are systemic, multi-dimensional and multi-organ, including skeletal muscle, cardio-respiratory system, vascular system, liver, fat, and brain. Hormones, exerkines, and metabolites produced by different tissues, and organs during and after exercise are secreted into the blood circulation and then act on brain tissue, thereby playing a neuroprotective role. The discovery of novel mediators associated with exercise and their mechanism of action will be an important development direction in this field.

Since Berzelius first discovered the presence of free lactic acid in muscles in 1808, lactate has attracted much attention in the field of sports science. It was previously believed that lactate produced by skeletal muscle contraction during exercise was a "waste" and an important trigger of muscle fatigue. Simultaneously, the theory of oxygen debt and the concept of anaerobic threshold were put forward, which was regarded as the classical era of lactate research. With the advent of new technologies and new experimental results, especially Brooks et al. systematically proposed the theory of intercellular lactate shuttle in 1985 as well as the hypothesis of intracellular lactate shuttle in 1998 [1, 2], there is a new understanding of the role of lactate, i.e. lactate is not a direct cause of fatigue but a biomarker; it is not a cause of limiting maximal oxygen uptake during incremental exercise, nor is it a cause of metabolic end-waste, but rather a metabolite intermediate and an important energy substrate [3]. This breakthrough understanding has brought new opportunities for research in the post-lactate era.

In recent years, research on lactate has been increasing in neuroscience and expanded the understanding that lactate is regarded as the mediator of the effect of exercise on brain health. Studies have shown that lactate increases the transcription and protein levels of brain-derived neurotrophic factor (BDNF) in neurons and glial cells. Lactate produced by astrocyte "aerobic glycolysis" plays a crucial role in memory acquisition in mice and maintains learning-dependent synaptic plasticity [4]. Inhibition of lactate production using dichloroacetate, mice showed impaired memory in water maze experiments. Besides, a certain intensity of exercise can promote brain uptake of skeletal muscle-derived lactate and enhance aerobic glycolysis to increase brain-derived lactate production: on the one hand, during moderate or high-intensity exercise, skeletal muscle releases lactate into the blood circulation, resulting in increased blood lactate levels and proportional brain uptake of myogenic lactate; on the other hand, enhanced neuronal activity during exercise leads to increased levels of neuroactive substances, and neurons release more glutamate into the synaptic cleft, which stimulates glycogen degradation and aerobic glycolysis in astrocytes, respectively. This can result in increased brain-derived lactate production during exercise. It should be noted that this lactate modulates cognitive function mainly through two different mechanisms: as an energy substrate for neurons to meet a huge amount of energy demand generated from increased synaptic activity; as a signaling molecule to activate specific receptors to initiate plasticity-related signal transduction pathways. Therefore, the primary goal of this review article is to focuses on the neuroprotective role of lactate in the mechanism of exercise to improve cognitive function, in order to confirm the beneficial effect of exercise intervention on the achievement of healthy aging.

## Lactate as an energy supply substrate to maintain neuronal activity

### Astrocyte and lactate: new insights into brain energy metabolism

Although the human brain accounts for only 2% of the total body weight, it constitutes approximately 15% of the total blood flow provided by the cardiovascular system. When in the resting state, the brain utilizes at least 25% of the body's circulating glucose and about 20% of total body oxygen to maintain neuronal activity [5], suggesting a high energy demand of the brain and the importance of adequate energy supply to proper brain function. Glucose is an essential substrate for brain energy metabolism. However, several studies have demonstrated that lactate is a preferential energy substrate for neurons under some conditions. For example, neurons decrease glucose uptake in primary cultured cortical neurons exposed to glutamate [6]. In the cerebellum, glial cells rather than neurons absorb a large amount of glucose [7]. When the neurons are firing, the contribution of lactate to brain metabolism ranges from 7 to 60% [8, 9]. Surprisingly, most of the glucose consumed by neurons appears to be metabolized through the pentose phosphate pathway to maintain antioxidant status rather than participate in energy metabolism. Moreover, other studies have shown that 75% of oxidative metabolism in neurons is supported by lactate [10, 11]. Therefore, lactate may be an important energy supply substrate for the brain, especially when neuronal activity is enhanced.

The high demand for energy in the brain requires that the supply and utilization of energy substrates must be fine-tuned for specific spatiotemporal patterns based on changes in neuronal activity levels. This is mainly regulated by two aspects: on the one hand by regulating local cerebral blood flow (also known as neuro-vascular coupling), and on the other hand by regulating the shuttle and metabolic pathways of energy substrates between astrocytes and neurons (also known as neuro-metabolic coupling). Consistent with the latter situation, over the past decade, our understanding of brain energetics has rapidly evolved from a "neuron-centric" perspective into a "tight coupling between the neuronal and astrocyte processes" [12]. This presents a new research paradigm emphasizing the importance of astrocytes in brain energy metabolism. In fact, astrocytes are the most abundant glial cells in the brain, accounting for about 30% of the brain volume, potentially ten times higher than neurons. Besides, astrocytes have a large number of radial end feet, some of which wrap around cerebral microvasculature to form the blood–brain barrier, and others connect with axons or dendrites of neurons. This unique anatomical structural feature determines that astrocytes provide a bridge between neuronal activity and vascular blood circulation, thus playing an essential regulatory role in the ingestion of substances from the blood circulation, information transmission, and energy supply [13, 14].

### Astrocyte-neuron lactate shuttle (ANLS)

**Fig. 1:**
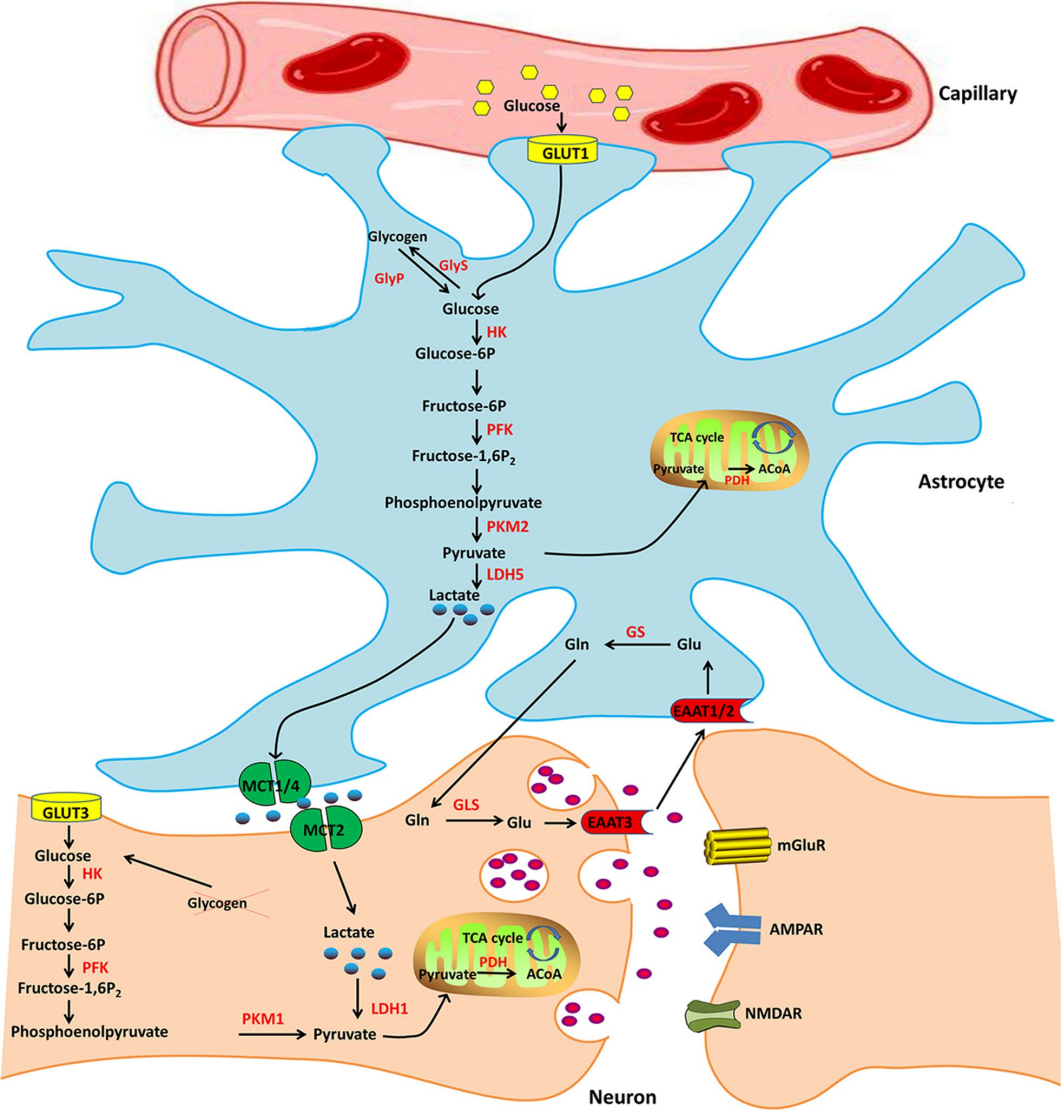
Schematic diagram of astrocyte-neuron lactate shuttle (ANLS). ACoA, acetyl-CoA; EAAT, Excitatory amino acid transporter; HK, Hexokinase; Gln, Glutamine; GLS, Glutaminase; GLUT, Glucose transporter; GlyP, Glycogen phosphorylase; GlyS, Glycogen synthase; GS, Glutamate synthase; LDH, Lactate dehydrogenase; MCT, Monocarboxylic acid transporters; PDH, Pyruvate dehydrogenase; PFK, Phosphofructokinase; PKM1, Pyruvate kinase M1; PKM2, Pyruvate kinase M2; TCA cycle, Tricarboxylic acid cycle

Circulating glucose can enter the brain parenchyma via glucose transporter1 (55 kDa) in cerebrovascular endothelial cells. There are different subtypes of GLUTs in astrocytes and neurons, which uptake glucose through GLUT1 (45 kDa) and GLUT3, respectively. Although both astrocytes and neurons can consume glucose to produce ATP through glycolysis and tricarboxylic acid (TCA) cycle, they have different expression and activity of the key metabolic enzymes, resulting in displaying different metabolic phenotypes. Pyruvate kinase M2 (PKM2) and lactate dehydrogenase 5 (LDH5) are predominantly expressed in astrocytes, and the activity of 6-phosphate fructose kinase 1 (PFK-1) in astrocytes is stronger, which indicates stronger glycolysis ability. By comparison, PKM1 and LDH1 are mainly expressed in neurons, and the activity of pyruvate dehydrogenase (PDH) is higher [15]. Thus, neurons exhibit the enhanced capability to conduct the TCA cycle. Moreover, research showed that glutamate could stimulate astrocytes to ingest glucose and enhance their glycolytic activity, leading to increased levels of extracellular lactate. Based on the findings above, Pellerin and Magistretti proposed the astrocyte-neuron lactate shuttle (ANLS) hypothesis in 1994 [16]. The core content is that neurons can take up extracellular lactate as an essential supplement in energy metabolism substrate, while astrocytes provide small molecular energy substances to the neurons wrapped around them by releasing lactate (Fig. 1). Specifically, ANLS initiates from neuronal activity. When neuronal excitability is enhanced, glutamate is released from the presynaptic membrane into the synaptic cleft, which then stimulates the uptake of circulating glucose in astrocytes through GLUT1. Glutamate can be transported to intracellular space by excitatory amino acid transporters (EAATs) expressed in astrocyte membranes. Simultaneously, more Na + enters intracellular space, and then the sodium–potassium pump (Na^+^-K^+^ ATPase) is activated to expel Na^+^, which leads to increased adenosine triphosphate (ATP) consumption and adenosine monophosphate (AMP)/ATP ratio in astrocytes. The result is glycolytic enzymes, such as hexokinase (HK) and PFK being activated, leading to enhanced glycolysis in astrocytes. The produced lactate is transported to the intracellular space via monocarboxylic acid transporter (MCT) 1/4 expressed in the astrocytes membrane, and then taken up into neurons by MCT2 expressed in the neuronal membrane, thus enabling the shuttle of lactate from astrocytes to neurons. Subsequently, by the action of LDH1, lactate in neurons is converted to pyruvate, which is further subjected to oxidative decarboxylation by PDH and transformed to acetyl-CoA.

Neurons produce ATP from pyruvate-derived acetyl-CoA through the TCA cycle. Glutamate, which can be transported from the synaptic cleft to astrocytes and triggers lactate shuttling, is partly converted to glutamine by the catalysis of glutaminase (GLS) and released to extracellular, and then absorbed by neurons and converted to glutamate under the catalysis of glutamate synthase (GS). Another part of glutamate is involved in the synthesis of reduced glutathione in astrocytes [17–19]. Therefore, ANLS can not only provide lactate for the TCA cycle of neurons, but also prevent excessive glutamate in the synaptic cleft leading to excitotoxic neuronal injury and produce antioxidants such as glutathione (GSH) to protect cells against oxidative stress. Taken together, neurons may regulate the energy supply for astrocytes to effectively improve the uptake, delivery, and utilization of energy substrates when synaptic activity is enhanced. Although neurons and astrocytes develop different metabolic phenotypes, both influence and regulate each other to maintain the homeostasis of brain energy supply together.

### Glycogen is an important energy reserve for the brain

Lactate in the brain can be generated through astrocytes uptake of circulating glucose and initiating glycolysis, as well as through neuromodulators/ neuroactive substances to promote glycogenolysis. The latter way, namely astrocytic glycogen-derived lactate, represents an alternative way of how ANLS operates. In the brain, glycogen is distributed almost completely in astrocytes and only found in the brainstem, choroids plexus, and ependyma in small quantities [20, 21]. This is because glycogen and glycogen phosphorylase (GlyP), a key enzyme involved in the glycogen breakdown, specificity exists in astrocytes. Neuron-expressed glycogen synthase (GlyS) is continuously degraded via the ubiquitin–proteasome pathway, and there is a lack of glycogen phosphorylase that catabolizes glycogen in neurons, in order to prevent neuronal death caused by glycogen accumulation in neurons under pathological conditions [22–24]. Moreover, glycogen granules are preferentially located in the astrocyte processes surrounding synapses rather than randomly distributed in astrocytic cell bodies of the hippocampus and cortex [25, 26]. Therefore, when neuronal energy demand increases or energy substrates are insufficient such as hypoglycemia, cerebral ischemia and sleep deprivation, glycogen stored in astrocytes may provide a supplementary energy reserve that is rapidly metabolized to lactate, and then transported to neurons which are used as an energy substrate, thus coupling transient energy demand generated by synaptic activity. In short, glycogen is a dynamic component of brain energetics and can be selectively used to promote memory consolidation, even in the presence of glucose. Astrocytic glycogen-derived lactate is critical for neural plasticity and memory.

### Monocarboxylic acid transporters are key molecules in lactate shuttle

Lactate requires specific transporters to be released and taken up by different types of cells. The Monocarboxylic acid transporters (MCTs) family members, MCT1-4, are mainly responsible for transmembrane transport of various monocarboxylic acids such as lactate, short-chain fatty acids, and ketone bodies, and play an important role in the intercellular and interorganizational transport of lactate [27]. Therefore, MCTs are key participants in the lactate shuttle. Studies have uncovered three MCTs subtypes in the central nervous system, with clear cellular distribution in the brain. Concretely, MCT1 is expressed in astrocytes, microvascular endothelial cells, ependymal cells, and oligodendrocytes, and acts as carriers for lactate to cross the blood–brain barrier, thus playing an essential role in the entry of circulating lactate and ketone bodies into the brain. MCT4 is mainly expressed in astrocytes with high glycolytic activity and the production of large amounts of lactate. Therefore, MCT1 and MCT4 participate in both inward and outward lactate transport of astrocytes. In addition, since the Km of MCT1 for lactate is lower than that of MCT4 (3.5 vs. 34.7 mM, respectively) [28], the specific role of these two transporters varies depending on the status of producing or releasing lactate in astrocytes. MCT2 is mainly expressed in neurons with highly oxidizing ability and is responsible for the uptake of extracellular lactate. MCT2 has a Km for lactate of less than 1 mM [29, 30]. With a difference in lactate concentration inside and outside the cell, MCT2 located in the membrane of neurons transports lactate from the extracellular space into the cell along the concentration gradient. The distribution of MCTs is consistent with the concept of the astrocyte-neuron lactate shuttle.

Moreover, the expression and the location on the plasma membrane of MCTs in the brain showed changes in response to hypoxia, intense aerobic exercise, ischemia, and prolonged fasting [31], and also seem to be susceptible to various signals emerged from neuronal activity as well as a variety of neuroactive substances, such as Noradrenaline, insulin, insulin-like growth factors and BDNF [32–35]. It is speculated that compared with glucose, monocarboxylic acids are more adaptable as a neural energy substrate during neuronal activity levels change [28].

### ANLS and cognitive function

As is well known, the central nervous system requires high energy levels to maintain various physiological activities, especially in the processes of neural plasticity and memory formation. Brain energy metabolism disorders may affect the transmission of synaptic information, and cause a series of pathological changes such as neuronal loss and degeneration, further damaging cognitive function.

ANLS is capable of meeting the additional energy demands generated from enhanced synaptic activity and then participates in the process of plasticity, including cytoskeleton rearrangement, up-regulation of gene expression, and protein transport [36]. Therefore, impairment of the astrocyte-neuron lactate shuttle is an important contributor to cognitive deterioration. The autopsy results have shown that the activity of LDH5 [37] in the brain reduces and the expression of GLUT1 [38] which is expressed in endothelial cells and astrocytes decreases in patients with cognitive impairment. Young adult apoE4 carriers had a higher genetic risk of late-onset AD, with increased levels of MCT2 and decreased levels of MCT4 in the posterior cingulate of the limbic system [39]. Animal studies showed that both astrocytic EAAT1/2 level and neuronal MCT2 level were significantly reduced in an AD mouse model [40]. Treatment of hippocampal slices with 4-CIN, an inhibitor of MCT2, can inhibit lactate uptake by hippocampal neurons and exacerbate glutamate-induced neuronal discharge abnormalities in the hippocampal CA1 region [41]. In a rat model of AD, bilateral hippocampal injection of amyloid beta-peptide 25–35 fragments significantly decreased lactate content and MCT2 expression, accompanied by memory and learning deficits [42]. The above studies suggested that the abnormality of key players in ANLS may represent a critical pathologic mechanism of cognitive dysfunction in AD. In addition to AD, some progress has been made in understanding the pathogenesis of other cognitive disorders. Mice with long-term ketamine administration showed dose-dependent learning and memory impairment as well as lower hippocampal MCT1 and MCT4 membrane protein levels and higher cytoplasmic protein levels; both MCT2 protein and mRNA levels in the hippocampus were significantly increased, suggesting that long-term ketamine administration-induced cognitive impairment may be related to abnormal expression of MCTs in the hippocampus [43]. It has also been reported that the MCTs inhibitor 4-CIN can significantly reduce the immobility time during fear contextual conditioning training, suggesting that MCTs are indispensable in the acquisition of amygdala-dependent contextual fear memory [44]. Knockdown of Astrocytic Monocarboxylate Transporter 4 in the Motor Cortex resulted in worse motor performance and learning, reduced dendritic spine density and decreased plasticity-related protein expression [45]. Besides, lactate shuttle mediated by MCT1 and MCT2 is also necessary for the reward memories for relapse in cocaine addiction [46]. Taken together, abnormal expression of key players in ANLS including key metabolic enzymes and transporters can affect the lactate shuttle, and thus play a negative role in learning and memory.

In addition, inhibition of lactate produced by astrocytic glycogenolysis and the transfer of lactate from astrocytes to neurons can impair memory processes in rodents. Studies in 1-day-old chicks led by Gibbs et al. [47] revealed a reduction in brain glycogen content during cognitive training, and inhibition of glycogenolysis in astrocytes interrupted memory consolidation in chickens. Suzuki et al. [48] found that bilaterally injection of the inhibitor of glycogenolysis1,4-dideoxy-1,4-imino-D-arabinitol (DAB) blocked long-term potentiation (LTP) and long-term memory in inhibitory avoidance task, with concomitant downregulation of plasticity-related molecules including Arc, p-CREB, and p-CFL1. This effect could be reversed by joint injections of DAB and lactate. Similarly, down-regulation of astrocytic lactate transporters MCT1 or MCT4 by intra-hippocampal oligodeoxynucleotide injections could also disrupt long-term memory formation and be reversed by exogenous supplementation of L-lactate; in contrast, injection of exogenous lactate or glucose could not counteract cognitive impairment resulting from inhibition of neuronal lactate transporter MCT2 expression, implying that MCT2-mediated transport of lactate to neurons is required for LTP and long-term memory formation.

## Lactate acts as a signal molecule regulating cognitive-related molecular mechanisms

### The lactate receptor GPR81 /HCAR1

Lactate acts as a signaling molecule to modulate plasticity via the activation of specific receptors and subsequent downstream signaling pathways. Currently, a specific receptor for lactate has been identified and named G protein-coupled receptor 81 (GPR81), also called Hydroxycarboxylic acid receptor 1 (HCAR1). As a member of the hydroxycarboxylic acid receptor family, it is activated over a range of physiological lactate concentrations (1 ~ 20 mmol /L) and EC50 values for specificity ligand lactate are between 1 and 5 mM [49]. HCAR1 exerts biological functions in the body including inhibiting lipolysis, inhibiting inflammatory response, regulating mitochondrial ATP synthesis, and modulating cerebral blood flow. Studies have found that HCAR1 is widely distributed in the adipose tissue, placenta, brain, skeletal muscle, bone, and heart. Among them, the distribution of brain HCAR1 in mice shows specific regularity. It is highly expressed in cerebellar Purkinje neurons and dendrites, hippocampal dentate granule cells, dentate gyrus neurons, and neocortical neurons. The colloidal gold immunoelectron microscopy quantitative analysis showed that a high immunoreactivity for HCAR1 was observed at the excitatory postsynaptic membrane of the pyramidal cell dendritic spines, and it was also expressed in endothelial cells and astrocyte end-feet of the blood–brain barrier as well as synaptic structure wrapped around astrocytes [50]. All the above suggested that lactate receptor HCAR1 plays a role in a wide range of regions of the central nervous system, which may mediate the regulation of lactate on synaptic function, energy metabolism, and cerebral blood flow, and ultimately affect learning and memory ability.

It should be noted that the HCAR genes are located on the short arm of human chromosome 12 (12q24.31) which also contains neuron-specificity MCT2 (12 q.13). Lactate produced by enhanced neuronal activity is transported to neurons through MCT2 subsequently activates the co-localized lactate receptor, showing that MCTs and HCAR1 involve the coordinated regulation of lactate in the brain. Moreover, both HCAR1and MCT2 have the highest concentrations in pyramidal neurons, and show dual co-localization to the synaptic membrane and vesicular organelles, suggesting a new possibility to regulate lactate signaling, by the turnover of lactate receptor and lactate transporters between the synaptic membrane and intracellular vesicular organelles [51].

### Lactate serves as a signal molecule to activate the intracellular signal transduction pathway

**Fig. 2:**
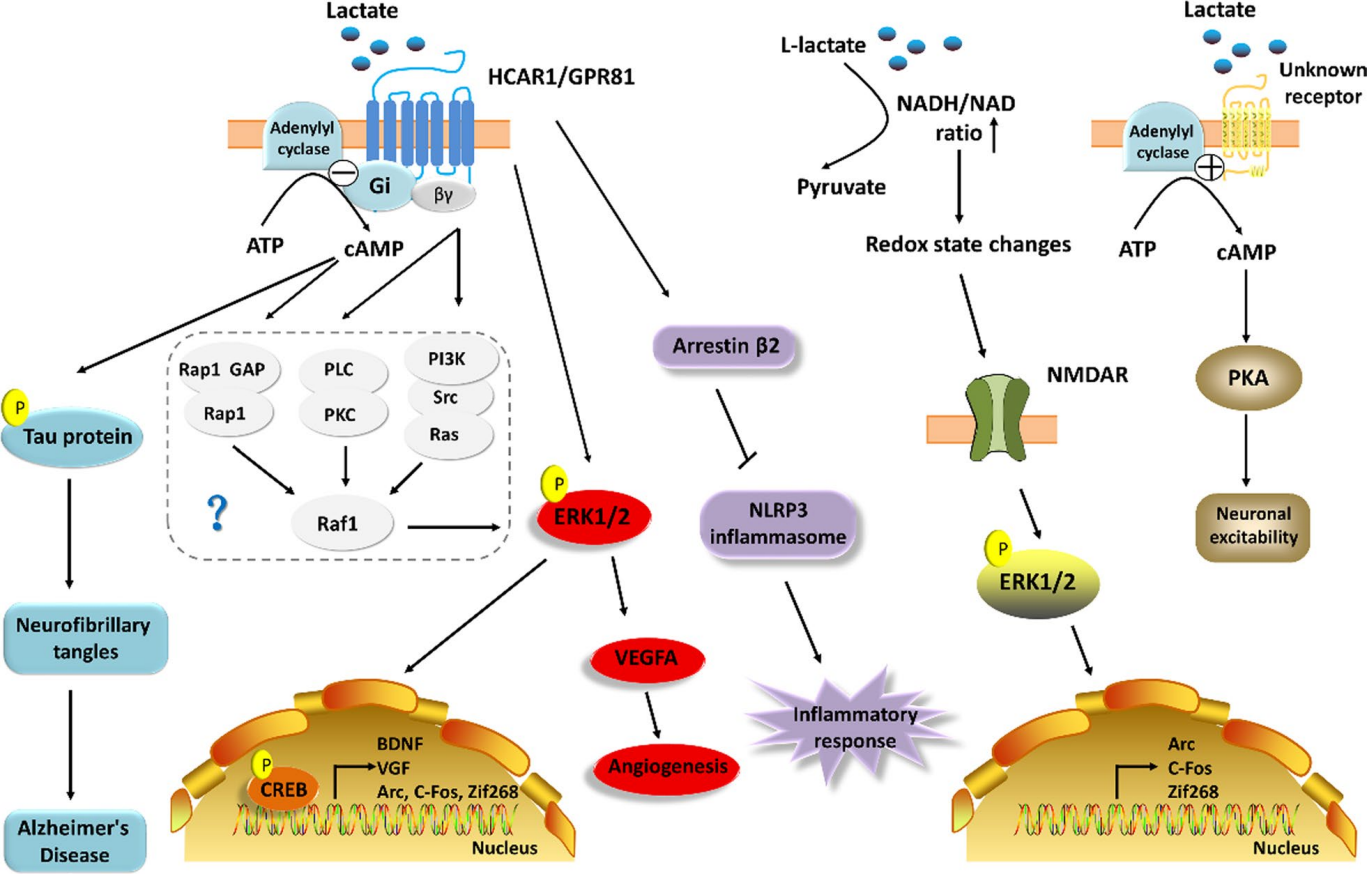
Putative mechanisms of lactate as a signal molecule to regulate cognition. ATP, Adenosine triphosphate; BDNF, Brain-derived neurotrophic factor; cAMP, Cyclic adenosine mono-phosphate; CREB, Cyclic AMP-responsive element-binding protein; ERK, Extracellular regulated protein kinases; Gi, Inhibitory adenylate cyclase G protein; GPR81, G protein-coupled receptor 81; HCAR1, Hydroxycarboxylic acid receptor 1; NLRP3, NLR family pyrin domain containing 3; NMDAR, N-methyl-D-aspartic acid receptor; PKA, Protein kinase A; VEGFA, Vascular Endothelial Growth Factor A; VGF, VGF nerve growth factor inducible

Studies have confirmed the vital role of lactate in regulating plasticity, angiogenesis, and neuronal excitability, ultimately impacting synaptic transmission and function. The process is achieved by binding lactate to lactate receptor HCAR1, N-methyl-D-aspartic acid receptor (NMDAR), and an undetermined G protein-coupled receptor (Fig. 2).

Previous works have proven that lactate/HCAR1 can activate multiple signal transduction pathways, thus regulating cognitive-associated molecular. Multiple extracellular signals are known to activate extracellular signal-regulated kinase (ERK) 1/2 by G protein-coupled receptors. Concretely, the α subunit of Gi can attenuate Rap1 suppression of the Raf1 via a reduction in cyclic adenosine monophosphate (cAMP) levels and the recruitment of GTPase-activating proteins, thereby increasing the phosphorylation level of ERK1/2; The βγ subunits released from activated Gi can increase the ERK1/2 phosphorylation through PLC-PKC-Raf1 and PI3K-Src-Ras-Raf1 signaling pathway [52]. The phosphorylation of ERK1/2 may activate cAMP response element-binding protein (CREB) through up-regulation of MSK/p90RSK, which in turn alters the expression of plasticity-related genes including immediate early genes (IEGs), BDNF, and VGF nerve growth factor inducible (VGF). Since HCAR1 belongs to the inhibitory G protein-coupled receptor family, it is likely that HCAR1 influences neural plasticity at least partly through an up-regulation of ERK1/2 phosphorylation in neurons. Morland et al. [53] found that subcutaneous injection of lactate in wild mice for 7 weeks could elevate blood lactate levels up to ~ 10 mM, also increase capillary density in the sensorimotor cortex and the dentate gyrus of the hippocampus, with a concomitant increase in hippocampal VEGF protein expression, while these beneficial effects disappeared in HCAR1 knockout mice. Further studies found that either lactate or HCAR1 agonist 3,5-dihydroxybenzoate (3,5-DHBA) increased ERK1/2 and Akt phosphorylation levels in hippocampal slices of wild-type mice but not HCAR1 knockout mice. This suggests that lactate/ HCAR1 in the pia and/or brain upregulates vascular endothelial growth factor A (VEGFA) and angiogenesis by enhancing phosphorylation of ERK1/2 and Akt, and it eventually leads to an increase in cerebrovascular density. Interestingly, this study also reported that no HCAR1-dependent angiogenesis in peripheral organs, such as in the liver or in muscles. It is known that excessive cAMP levels in the prefrontal cortex are associated with age-related cognitive decline [54]. Lactate reduced the detrimental effects of long-term elevated cAMP levels by binding to and activating HCAR1, for example, it could prevent neuronal overactivity and regulate cAMP-activated potassium channel activity to counteract excessive hyperpolarization [55]. Both HCAR1 agonists 3,5-DHBA and the physiological concentration of lactate were found to downregulate abnormally elevated cAMP levels stimulated by forskolin in hippocampal slices [50]. The activation of HCAR1 may counteract elevated levels of phosphorylated tau induced by cAMP, which causes impaired synaptic function and the deposition of neurofibrillary tangles, and eventually slowing down the course of AD. Reduced neurological deficits was observed by lactate pretreatment in a traumatic brain injury rat model. Lactate pretreatment also upregulated the expression of GPR81, MCT2 and plasticity-related proteins (PSD95, GAP43, BDNF) in ipsilateral cortex and ipsilateral hippocampus. This suggests that targeting lactate/GPR81 signaling pathway may provide a potential therapy for traumatic brain injury [56]. Moreover, as with other G protein-coupled receptors, HCAR1 may activate intracellular non-classical G protein-independent signaling. For example, the HCAR1-arrestin β2-NLRP3 pathway mediated a neuroprotective effect of ketogenic diets in glaucoma by suppressing inflammatory response in the glaucomatous retina and optic nerve [57]. However, the anti-inflammatory action of HCAR1 remains to be confirmed in cognitive-related studies.

Lactate has also been reported to act on NMDA receptors to promote plasticity gene expression. In primary cultured mouse cortical neurons and in sensorimotor cortex in vivo, L-lactate induces the expression of immediate early genes such as Arc, c-Fos, and Zif268, which are considered key genes involved in neural plasticity and maintenance of CNS function. Further studies show that this role of L-lactate in neurons is mediated by regulating NMDA receptor activity and its downstream Erk1/2 signaling cascades, as well as the regulation of the cellular redox status of neurons [58]. Therefore, these results confirm that lactate acts as a brain signaling molecule to activate a previously undetected receptor, the NMDA receptor, to regulate neural plasticity.

In addition, there are other receptors for lactate. Tang et al. [59] found that L-lactate released by astrocytes could not be used as an energy source by noradrenergic neurons in the locus coeruleus, but instead bind to a currently unknown receptor to active noradrenergic neurons. This process was dependent on an increase in cAMP levels and positive regulation of AC. Notably, this putative lactate receptor is distinct from previously described HCAR1, because HCAR1 belongs to Gi-coupled receptors which can inhibit neuronal excitability. Since the locus coeruleus nucleus is associated with hippocampal memory consolidation, it is speculated that lactate binds to an undetermined G protein-coupled receptor to play a role in learning and memory.

## The potential mechanisms of lactate-mediated exercise-induced improvements in cognitive function

Accumulating evidence suggests that moderate-to-vigorous-intensity physical activity is positively correlated with a lessened risk of cognitive impairment and dementia. Remarkable progress has been achieved in the mechanism understanding of exercise-enhanced cognitive function within the last two decades, and this has been elaborated in a number of excellent reviews [60–63]. Regular exercise can enhance neurogenesis and synaptic plasticity, improve cerebral hemodynamics, increase the expression of neurotrophic factors, attenuate central inflammatory response, and subsequently have a positive effect on cognitive function and brain health. Some studies have confirmed that lactate and its receptor serve as potential targets for the treatment of age-related cognitive impairment. It is well known that moderate to high-intensity exercise significantly increases the level of lactate in the blood and brain. In particular, recent findings indicated that lactate-generated from exercise slows down the pathological progression of brain aging and neurodegenerative diseases. Therefore, lactate can be an important mediator of exercise to promote brain health.

### Lactate acts as an energy source for the brain in exercise to improve cognitive function

#### The increased amount of lactate during exercise can provide energy to the brain

**Fig. 3:**
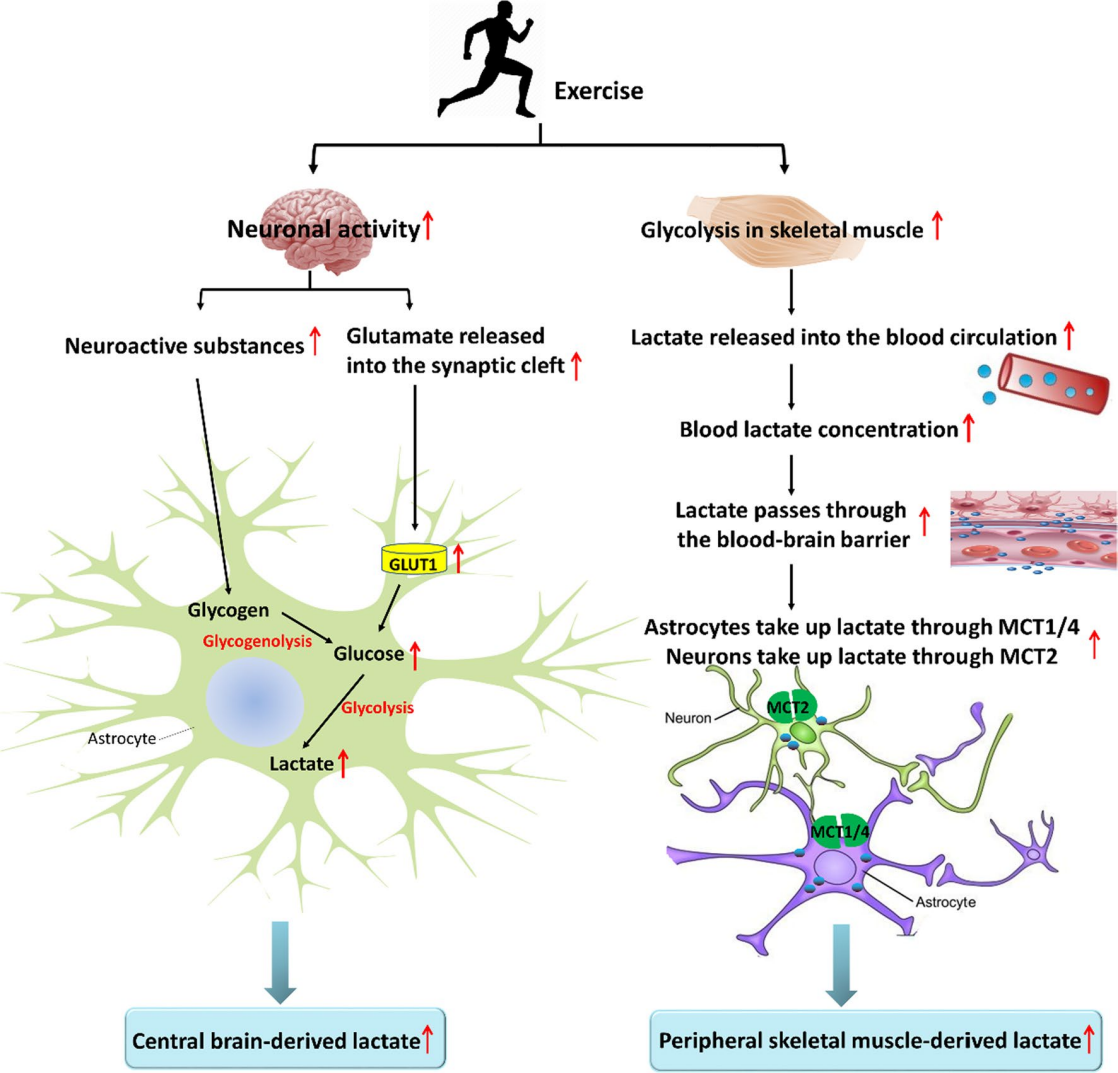
Exercise increases brain lactate levels in two ways, including central brain-derived and peripheral skeletal muscle-derived. GLUT 1, Glucose transporter 1; MCT, Monocarboxylic acid transporter

Exercise increases brain lactate levels in two ways, namely, central brain-derived and peripheral skeletal muscle-derived, which respectively regulate brain functions in a hormone-like "paracrine" manner and "distant secretion" manner (Fig. 3). Specifically for central brain-derived lactate, exercise firstly increases the secretion of neurotransmitters and neuromodulators in the brain such as glutamate, norepinephrine, vasoactive intestinal peptide, pituitary adenylate cyclase-activating peptide, and serotonin [64, 65], which can trigger glycogenolysis in astrocytes. The role of these neurotransmitters and neuromodulators is well known in the modulation of memory processes, speculating that some neurotransmitters can participate in the learning and memory process via promoting the lactate-derived from glycogenolysis in astrocytes [66]. Secondly, the brain is activated during exercise, which stimulates the release of glutamate from pre-synaptic terminals to the synaptic cleft, resulting from increased excitability of neurons. Then GLUT1 expression is significantly up-regulated to stimulate circulating glucose uptake by astrocytes and initiates anaerobic glycolysis to generate lactate, completing the transfer of lactate from astrocytes to neurons. These two ways represent a mechanism by which exercise increases the formation of brain-derived lactate. Moreover, the lactate produced by skeletal muscle during exercise can be released into the blood circulation, and the net transport of lactate across the blood–brain barrier is inwards around the situation [67]. Lactate passes through the blood–brain barrier by passive transport mediated by MCT1, and subsequently enters the brain parenchyma. Uptake of blood lactate by the brain increases in proportion to the intensity of exercise. More specifically, in the quiet state, human blood lactate concentration is about 0.5–2 mM, and brain lactate concentration ranges from 0.1 to 1 mM. However, during progressive increasing load volume training, skeletal muscle shows enhanced glycolysis, leading to a gradual increase in blood lactate concentration with up to 20–30 mM. At that time, part of blood lactate is converted to glucose via hepatic gluconeogenesis or enters amino acid metabolic pathways under the action of transaminase. Another part of blood lactate is transported into the brain through MCTs, with a rise of brain lactate concentration close to 2 mM [51, 68]. Cerebral lactate uptake during exercise is directly proportional to arterial lactate concentration, and is decreased in the last 2 min of early recovery and shows a linear relationship at the final stage of recovery. Furthermore, when the concentration of lactate is between 1 and 15 mM, there is no indication that cerebral lactate uptake will be saturated [67], indicating that the brain is with the high-capacity lactate transport system.

So, can the increased lactate in the brain participate in brain energy metabolism during exercise? Smith et al. [69] found that lactate almost did not participate in the brain energy supply in the quiet state, with glucose and oxygen accounting for 51.7% and 48.3% of the cerebral metabolism rate (CMR), respectively. When exercise intensity reached 60% maximal oxygen uptake (VO_2_max), cerebral lactate uptake significantly increased, and the proportion of lactate in the CMR could reach up to 10.6%. As exercise intensity further increased, cerebral lactate uptake continued to increase, and the contribution to CMR of lactate was also greater. When exercise intensity increased to VO_2_max, the proportion of lactate in CMR increased to 27.8%, while the contribution rate of glucose and oxygen to CMR decreased to 38.6% and 33.6%, respectively. Compared to pre-exercise, there was a ∼tenfold increase in human cerebral lactate uptake in response to exhaustive exercise. This trend could continue even for a period time after cessation of exercise. The increased amount of cerebral uptake of lactate far exceeded glucose and oxygen [70]. This indicates that a high-intensity exercise-induced increase in blood lactate can contribute to brain energy supply and in a certain range, the greater the exercise load, the greater the contribution rate.

#### Exercise improves cognitive function by regulating ANLS-related metabolic enzymes and transporters

ANLS is required for memory formation and consolidation and can be a connecting link between exercise and neural plasticity. Based on the above-mentioned mechanism of ANLS, metabolic enzymes and transporters involved in the process are regarded as important regulatory targets. Exercise has been shown to regulate the expression or activity of ANLS-related molecules. MCTs mediate the transport of lactate from blood circulation to astrocytes as well as astrocytes to neurons, and thus being considered the important molecular basis for lactate to play a role during exercise. Both acute exercise and long-term exercise can modulate the expression of MCTs in the brain. Takimoto and Hamada [71] confirmed that increased expression of MCTs in brain cognition-related regions following a single acute bout of exercise. 2 h of moderate-intensity treadmill running could significantly increase the levels of lactate, MCT1, and MCT2 in the cerebral cortex and hippocampus of rats, and MCT2 expression was positively correlated with the up-regulation of neuronal BDNF and its receptor TrkB induced by exercise. Moderate-intensity treadmill running for four weeks resulted in increased glycogen level and MCT2 protein expression in hippocampal tissue as well as an improvement in spatial memory in diabetic rats [72]. Light and moderate intensity treadmill running for 4 months from the presymptomatic stage in Otsuka-Long-Evans-Tokushima fatty (OLETF) rats (a T2DM model) normalized MCT2, glycogen, and BDNF levels, accompanied by the improvement of hippocampal-dependent memory dysfunction, suggest that exercise can prevent the progression of T2DM-relative cognitive decline by targeting hippocampal lactate-transport [73]. 8-week swimming exercise significantly increased MCT1, MCT2, and MCT4 protein expression in brain tissue of wild mice and AD mice, accompanied by improvement in learning and memory abilities in the water maze test [74]. A 6-week high-intensity interval training (HIIT) significantly increased blood lactate and hippocampal lactate levels and promoted hippocampal ATP levels, MCT1/4 and BDNF expression. Simultaneously, mice of the high-intensity interval training group showed enhanced mitochondrial fusion and mitochondrial biogenesis in the hippocampus [75]. This indicates that HIIT modulates the hippocampal lactate transport and mitochondrial quality control systems to meeting the high energy requirements of the brain. Long-term moderate exercise could upregulate the expression of hippocampal BDNF, Trk B, and MCT4 in middle-aged mice, as well as the expression of glutamine synthase (GS) in the aged mice brain, and also rescued age-related decline in dendrite complexity and LTP of hippocampal CA1 neurons, and thereby restored learning and memory ability in middle-aged mice [76]. This further suggests that exercise can improve lactate transport in the brain, which is closely related to improving learning and memory ability. In a rat model of ischemic brain injury, pre-ischemic treadmill training for two weeks could up-regulate EAAT2 expression and remove excess glutamate from the synaptic cleft, in order to prevent excitotoxicity-induced by cerebral reperfusion, suggesting that exercise may exert neuroprotective effects by up-regulation of EAATs expression to maintain proper functioning of ANLS [77]. In addition, exercise also modulates GLUTs expression in the brain. The expression of astrocytic GLUT1 and neuroplasticity-related proteins in motor areas of the cerebral cortex was found to significantly increase following 48 h of voluntary exercise in mice [78]. 16-week treadmill exercise significantly reversed AD-associated decreased GLUT1 and BDNF expression, and reduced neuronal oxidative stress and excitotoxic injury by regulating superoxide dismutase1, H_2_O_2_, and Bcl-2 [79]. These findings suggest that exercise can provide sufficient glucose for astrocytic glycolysis by facilitating glucose transport, and then modulating oxidative stress as well as neural plasticity.

Interestingly, both hyperpalatable diet and physical exercise increased hippocampal MCT1 and MCT4 protein expression, but only exercise elevated the expression of PDH. PDH is the rate-limiting enzyme for pyruvate entering the TCA cycle, crucial for mitochondrial oxidative metabolism in neurons [80]. Factors other than lactate concentration, such as the capacity of neuronal mitochondrial oxidative metabolism, that is, the capability to use lactate to produce ATP, also play an important role. This is an essential factor in improving energy supply, which distinguishes exercise from other pathological states. Notably, one of the major causes of central fatigue induced by exhaustive exercise is the reduction of brain glycogen. Prolonged exhaustive exercise led to a depletion of muscle glycogen and reduction in ATP levels, without an increase in lactate levels in skeletal muscle. Correspondingly, in the brain, exhausted exercise not only increased the glutamine/glutamate ratio of the cerebral cortex and hippocampus but also enhanced protein expression of MCT2 and GLUT1, accompanied by glycogen levels decreased while lactate levels increased to maintain ATP levels. Further studies found that using glycogenolysis inhibitor DAB reversed the decrease in glycogen levels caused by exhaustion exercise, and both DAB and MCT2 inhibitors 4-CIN could reduce cerebral ATP levels and exercise endurance in rats [81, 82]. Taken together, these studies suggest that astrocytic glycogenolysis and lactate shuttle are essential for the maintenance of brain ATP levels and endurance capacity during exhaustive exercise, which may be a mechanism underlying the neuroprotective action.

### Exercise improves cognitive function by activating lactate and its downstream signal pathways

Lactate has a pleiotropic role in the body. It appears that lactate is not only an energy source/metabolite but also acts like a hormone with autocrine-, paracrine- and endocrine- like effects, as postulated by Brooks [83–85] as “lactormone” [86]. During intensive exercise, both central brain-derived and peripheral skeletal muscle-derived lactate may act as a signaling molecule, thus affecting neuroplasticity-related gene and protein expression in the brain [87]. Here, we discuss a hypothesis that lactate is a key factor for exercise-induced improvements in cognitive function as the “lactormone”.

As mentioned above, the concentration of lactate can reach 2 mM in the brain during moderate-intensity exercise. L-lactate can active HCAR1 with an EC50 of 1–5 mM [49, 88]. In other words, brain lactate concentration during exercise seems sufficient to active HCAR1. 7 weeks of HIIT was found to elevate blood lactate levels as well as hippocampal VEGFA levels, which played a role of lactate produced from exercise in promoting cerebral angiogenesis, which is mediated by activation of HCAR1 [53]. It is reported that VEGFA can not only stimulate angiogenesis [89] but also directly enhance neurogenesis and synaptic function [90]. A recent study conducted by the same research team [91] reported that a 7-week HIIT or intraperitoneal injection of L-lactate could significantly enhance neurogenesis in the ventricular-subventricular zone in wild type, but not in HCAR1 knock-out mice, suggesting that HCAR1 mediated the action of L-lactate. Further studies using primary cultures of leptomeningeal fibroblasts found that the activation of Akt/PKB occurs downstream of HCAR1 activation. The increase of p-Akt may lead to the upregulation of CREB and growth factors, which might counteract age-related cognitive decline. These results imply that HCAR1 is a potential target for exercise-induced improvement of cognitive decline, especially for brain diseases related to chronic cerebral hypoperfusion and microvascular dysfunction. Moreover, lactate released by the skeletal muscle during exercise could penetrate the blood–brain barrier and induce increased expression of BDNF and TrkB in the hippocampus, while enhancing hippocampus-dependent spatial learning and memory, which was mediated by Sirtuin1/PGC-1α/FNDC5 pathways in mice [92], thereby suggesting an alternative mechanism of exercise promoting brain health from an epigenetic perspective. Yang et al. [58] found that in neurons L-lactate modulated NMDA receptor activity, enhanced NMDA receptor-dependent inward current, and subsequent calcium influx, resulting in an increase in the expression of IEGs, including Arc, c-Fos, and Zif268. Previous research has confirmed that IEGs play a critical role in the maintenance of LTP and long-term memory. Regular exercise can increase the concentration of lactate in the brain and enhance synaptic structural plasticity and functional plasticity. Therefore, it is speculated that the molecular link between muscle activity and enhanced brain plasticity is likely mediated by lactate-initiated signal transduction cascade. However, the underlying mechanisms by which lactate as a signaling molecule affects exercise-induced improvements in cognition have not been elucidated completely. It could be a future direction for a subsequent study.

### Histone lactylation: could this be the future of exercise promoting brain health?

Lactylation is one of the most important ways to exert its biological functions of lactate, and is involved in a series of vital life processes, such as oncogenesis [93], embryonic development [94], uterine remodeling [95], immune disorders [96], macrophage polarization [97] and metabolic regulation. To date, only a limited number of studies have examined the role of histone lactylation in regulating central nervous system.

Studies have found that lactylation is widely present in the brain, and has been described to be present in many neural cell types, such as astrocytes, microglia, GABA-ergic and glutamatergic neurons. Lactate treatment caused an increase in lactylation of histones in primary cultured neurons, and 4-CIN (a MCT2 inhibitor) and MCT1/2 inhibitor inhibited lactate transport into neurons and inhibit increase in lactylation [98]. Lactate treatment also increased lysine lactylation immunoreactivity in the prefrontal cortex but not in the dentate gyrus of the adult hippocampus, suggest exogenous lactate stimulates protein lactylation in a cell-type-specific manner in the brain. In an in vitro model of high concentration of potassium-induced neuronal excitation, the lactate concentrations in culture supernatants significantly elevated, and the intracellular lactate levels increased in a time-dependent response trend. Further studies found that neuronal excitation was likely to stimulates protein lactylation through intracellular metabolism and the glycolytic pathway. Mice with electroconvulsive stimulation were used for in vivo experiments and results showed that both intracellular new lactate production and extracellular lactate uptake involved in neuronal activation-induced lactylation. Lysine lactylation immunoreactivity showed a significant positive relationship with c-Fos expression at the individual cell level. Moreover, both a single exposure to social defeat stress (SDS) and a chronic SDS for 10 consecutive days were reported to significantly increase c-Fos expression, lactate levels and lactylation levels in the prefrontal cortex, and the increase was attenuated by prior treatment with OX (LDH inhibitor) in mice with a single SDS [98]. A simple and multiple regression analysis showed that increased brain lactate levels is correlated with increased anxiety-like behavioral responses. The level of lysine lactylation correlated with c-Fos expression in mice received more social defeat stress. These results indicate that social defeat stress-associated neural excitation may elevate brain lactate levels and stimulate histone lactylation. This is directly relevant to decreased social behavior.

A recent study also found that histone lactylation regulated glycolysis-associated pathway and thus is critically involved in the pathology of Alzheimer disease. Specifically, in the case of AD, glycolytic pathways were highly active in microglia, which manifested as a significant increase in all Hif-1α, Pkm2 and Ldha expression [99]. During the process of glycolysis, a large amount of lactate was produced and promoted H4K12la modification, which may in return promote transcriptional level of several glycolytic genes in microglia, further enhancing glycolysis. A glycolysis/H4K12la/PKM2 positive feedback loop is thus formed. The interruption of this positive feedback loop by knocking out the Pkm2 gene may suppress the aberrant microglial activation in 5 × FAD mice, decrease inflammatory response in the brain. This process was accompanied by decreased Aβ plaque content and improvement of learning and memory [99]. This study provides new perspectives and an attractive target for early intervention of AD. However, to our knowledge, almost no studies have elucidated the association of exercise with histone lactylation in the brain. Whether lactate might be involved in regulating exercise-related cognition improvement by modulating histone lactylation remains to be determined.

## Conclusions and future perspectives

Human exercise training intrinsically increases energy expenditure and accumulates metabolic end-products due to nerve excitation and skeletal muscle contraction. Therefore, alterations in the brain and peripheral energy metabolism caused by exercise, may be a potential inducer of exercise impacting neuronal function. Moderate-to high-intensity exercise increases the formation of brain-derived lactate and myogenic lactate. The increased amount of lactate plays dual roles as an energy supply substrate and a signaling molecule in the brain and thus improves cognitive function. Concretely, enhanced neuronal activity during exercise stimulates astrocytic glycogenolysis and subsequent aerobic glycolysis. Exercise regulates the expression or activity of ANLS-related transporters and metabolic enzymes to meet the higher energy demand of neurons. Meanwhile, exercise affects the expression of plasticity-related genes through activation of an intracellular signaling cascade downstream of lactate, in order to counteract age-related cognitive decline. Redefining the perceptions of lactate may contribute to further revealing the underlying mechanism of promoting brain health by exercise.

Brain lactate homeostasis is regarded as one of the important neurobiological mechanisms of exercise preventing or delaying cognitive decline, however, some problems are worth further investigation. (1) MCTs and the capacity of mitochondria to oxidize lactate are involved in lactate transport and brain energy production respectively; hence, future studies need to explore the synergistic effect of MCTs and mitochondria function on maintaining brain energy homeostasis during exercise. (2) There may be more cell types of lactate shuttle in the nervous system, such as Schwann cells and oligodendrocytes, which also play an important role in neuronal function. The specific role of the lactate shuttle in different cell types during exercise remains to be further clarified. (3) Currently, the ANLS hypothesis is confronted with some challenges. A reverse shuttle of lactate from neurons to astrocytes has been proposed. However, controversies are most drawn not directly from experimental data but the theoretical computational models or stoichiometric metabolic models. Future studies should be designed to provide direct dynamic evidence with isotope labeling and new imaging techniques. (4) Since lactate receptor HCAR1 and MCT2 are co-localized within the neurons, future studies should be designed to explore in depth the interaction between them to identify novel molecular targets for drugs.

## Data Availability

Not applicable.

## Abbreviations

AD: Alzheimer’s disease
BDNF: Brain-derived neurotrophic factor
ANLS: Astrocyte-neuron lactate shuttle
GLUTs: Glucose transporter
TCA: Tricarboxylic acid
PKM1/2: Pyruvate kinase M1/2
LDH: Lactate dehydrogenase
PFK-1: 6-Phosphate fructose kinase 1
PDH: Pyruvate dehydrogenase
EAATs: Excitatory amino acid transporters
Na^ +^ -K^ +^ ATPase: Sodium–potassium pump
ATP: Adenosine triphosphate
AMP: Adenosine monophosphate
HK: Hexokinase
MCT: Monocarboxylic acid transporter
GLS: Glutaminase
GS: Glutamate synthase
GSH: Glutathione
GlyP: Glycogen phosphorylase
GlyS: Glycogen synthase
DAB: 1,4-Dideoxy-1,4-imino-D-arabinitol
LTP: Long-term potentiation
GPR81: G protein-coupled receptor 81
HCAR1: Hydroxycarboxylic acid receptor 1
NMDAR: N-methyl-D-aspartic acid receptor
ERK: Extracellular signal-regulated kinase
cAMP: Cyclic adenosine mono-phosphate
CREB: CAMP response element-binding protein
IEGs: Immediate early genes
VGF: VGF nerve growth factor inducible
3,5-DHBA: 3,5-Dihydroxybenzoate
VEGFA: Vascular endothelial growth factor A
NLRP3: NLR family pyrin domain containing 3
CMR: Cerebral metabolism rate
VO2max: Maximal oxygen uptake
HIIT: High intensity interval training
SDS: Social defeat stress
